# Draft genome sequences of eight bacteria isolated from the indoor environment: *Staphylococcus capitis* strain H36, *S. capitis* strain H65, *S. cohnii* strain H62, *S. hominis* strain H69, *Microbacterium* sp. strain H83, *Mycobacterium iranicum* strain H39, *Plantibacter* sp. strain H53, and *Pseudomonas oryzihabitans* strain H72

**DOI:** 10.1186/s40793-017-0223-9

**Published:** 2017-01-31

**Authors:** Despoina S. Lymperopoulou, David A. Coil, Denise Schichnes, Steven E. Lindow, Guillaume Jospin, Jonathan A. Eisen, Rachel I. Adams

**Affiliations:** 1 Department of Plant & Microbial Biology, University of California Berkeley, Berkeley, CA USA; 2 Genome Center, University of California Davis, Davis, USA; 3 CNR Biological Imaging Facility, University of California Berkeley, Berkeley, USA; 4 Department of Evolution and Ecology, University of California Davis, Davis, USA; 5 Department of Medical Microbiology and Immunology, University of California Davis, Davis, CA USA

**Keywords:** Built environment, Shower water, Airborne bacteria, Bacterial genomes

## Abstract

We report here the draft genome sequences of eight bacterial strains of the genera *Staphylococcus*, *Microbacterium, Mycobacterium, Plantibacter,* and *Pseudomonas*. These isolates were obtained from aerosol sampling of bathrooms of five residences in the San Francisco Bay area. Taxonomic classifications as well as the genome sequence and gene annotation of the isolates are described. As part of the “Built Environment Reference Genome” project, these isolates and associated genome data provide valuable resources for studying the microbiology of the built environment.

**Electronic supplementary material:**

The online version of this article (doi:10.1186/s40793-017-0223-9) contains supplementary material, which is available to authorized users.

## Introduction

Given that humans spend most of their lives in indoor environments [1], it is important to understand the microorganisms that can be found in these human-created structures. Previous work based on 16S rRNA gene surveys has described thousands of bacterial taxa from residences (e.g., [2]). Within these residences, periodically wet surfaces– such as shower walls, shower heads, sinks, drains – represent unique (compared to dryer areas within the home - [3–5]) and potentially medically important microbial communities [6]. Humans could readily interface with the microbial communities on these wet surfaces by direct contact or by inhalation from aerosolized particles. Focusing on these airborne microorganisms, Miletto & Lindow [7] collected aerosol particles from residences for genetic analysis and identified over 300 genera which they attributed to various sources including tap water, human occupants, indoor surfaces, and outdoor air.

An important tool in studying microbial communities involves culturing and genome sequencing. In order to expand our work on the microbiology of built environments [8] into a more experimental framework, we cultured bacteria from the air of residential bathrooms and report their genome sequences. Genome sequencing was utilized in order to provide insight into the basic biology of the bacteria collected in indoor environments and to aid with future metagenomic and transcriptomic efforts.

The eight isolates within five genera were isolated during a sampling campaign of residential bathrooms conducted in 2015. While simultaneously filtering aerosols for amplicon-based community composition analysis (which is in preparation and will be published elsewhere), petri dishes were exposed to the air to isolate viable bacteria. After an initial screening of multiple isolates by sequencing the full-length 16S rRNA gene and carrying out preliminary taxonomic classification, eight isolates were selected for further genomic sequencing based on an assessment of their putative importance in the built environment. Specifically, we favored strains that met the following criteria: they are commonly identified in indoor environments, they are likely inputs from a common source for indoor microbes (premise plumbing, outdoor origin [9]), and/or they (or their close relatives) can potentially impact human health. For instance, we include three species (four isolates) of staphylococci. CoNS are typically benign inhabitants of the human skin and mucous membranes, but they are associated with infections and can be pathogenic to humans with compromised immune systems [10]. *Mycobacterium iranicum* is a newly described species which has been isolated from clinical specimens originating in diverse countries including Iran, Greece, the Netherlands, Sweden and the USA [11], although genomic comparison indicated that this is likely an environmental bacterium [12]. *Pseudomonas oryzihabitans* (synonym *Flavimonas oryzihabitans*) has been isolated from water and damp environments such as rice paddies and sink drains [13]. The only two described species of the genus *Plantibacter*, *P. auratus* and *P. flavus*, have been detected as a tree endophyte [14] and a component from the phyllosphere of grass [15], respectively. Organisms within the genus *Microbacterium* belong to the class *Actinobacteria* in which some species are known for the production of a broad spectrum of secondary metabolites. The chemical ecology of microorganisms on indoor surfaces is a component of our ongoing research efforts in the built environment.

Here we report a summary classification and the features of these eight isolates collected as part of the Built Environment Reference Genomes initiative. Strains and their genomes have been deposited according to the following accessions: *Staphylococcus capitis* strain H36 (DSM-103511; GenBank ID LWCQ00000000), *S. capitis* strain H65 (DSM-103512; LWCP00000000), *S. cohnii* strain H62 (DSM-103510; LWAC00000000), *S. hominis* strain H69 (DSM-103553; LVVO00000000), *Microbacterium* sp. strain H83 (DSM-103506; LWCU00000000, *Pseudomonas oryzihabitans* strain H72 (DSM-103505; LWCR00000000), *Mycobacterium iranicum* strain H39 (DSM-103542; LWCS00000000), and finally *Plantibacter* sp. strain H53 (DSM-103507; LWCT00000000).

## Organism information

### Classification and features

Two growth media were used for the initial isolation of bacteria: lysogeny broth agar (LB, Difco Laboratories, Detroit, MI) and R2A agar (Difco Laboratories, Detroit, MI). Petri dishes were exposed to residential bathroom air for 1 h; 30 min during which shower water was running to create shower mist and 30 min after the shower was turned off. Petri dishes were mounted on vertical surfaces (door, wall, cabinets) at a height of approximately 1.50 m. Petri dishes were brought back to the laboratory, where LB plates were incubated at 28 °C for 48 h, and R2A plates were incubated at 28 °C for 5 days and at 35 °C for 3 days. Except for *Staphylococcus hominis* strain H69, which was isolated on LB agar medium at 35 °C, all other strains were isolated on R2A medium (Additional file 1). Research was approved by the University of California Committee for the Protection of Human Subjects Protocol ID 2015-02-7135, and the sampling was conducted in March, 2015.

Taxonomic classification of these isolates was undertaken after genome sequencing, either using the full-length 16S rRNA gene sequences or a concatenated marker gene approach. For *Microbacterium*, *Mycobacterium*, and *Plantibacter* there were insufficient publicly available genome sequences of close relatives for a concatenated marker approach. In these cases, the full length 16S rRNA gene sequence was uploaded to the Ribosomal Database Project [16] and added to alignments containing representatives of all close relatives (as estimated from BLAST [17]). These alignments were downloaded, cleaned with a custom script [18], and an approximately maximum likelihood tree was inferred using the default setting in FastTree [19]. Outgroups for all trees were type strains of another genus or genera within the same family. The sequence alignments supporting the phylogenetic trees of this article are available in the FigShare repository [20].

All strains were given a specific identifier (e.g., H83) based on our internal culture collection. The 16S rRNA gene trees for both *Microbacterium* and *Plantibacter* genera were poorly resolved (e.g., low bootstrap values), and these isolates were placed into polyphyletic clades with respect to the names of taxa in the genera (Additional files 2 and 3). In addition, while *Microbacterium* sp. H83 falls within a clade that contains mostly *M. foliorum*, this name also occurs outside the clade. Therefore we have not attempted to assign these isolates to a particular species. On the other hand, the rRNA gene for one isolate is found in a monophyletic clade with other *M. iranicum* isolates (Additional file 4) and thus we have assigned this the name *M. iranicum* H39, For the *Pseudomonas* and *Staphylococcus* isolates, the 16S rRNA gene trees were inadequate for taxonomic classification at the species level, but the genomes of numerous sequenced representatives of close relatives were available for further analysis. All available genome sequences of close relatives (to a max of 20 randomly selected genomes per species) were downloaded from NCBI. The file names and sequences were reformatted for easier visualization. The assemblies were then screened for 37 core maker genes [21] using PhyloSift [22] in search and align mode using “isolate” and “besthit” flags. PhyloSift concatenates and aligns the hits of interest so the sequences are subsequently extracted from the PhyloSift output files and added to a single file for tree-building. An approximately maximum-likelihood tree was then inferred using FastTree.

**Fig. 1:**
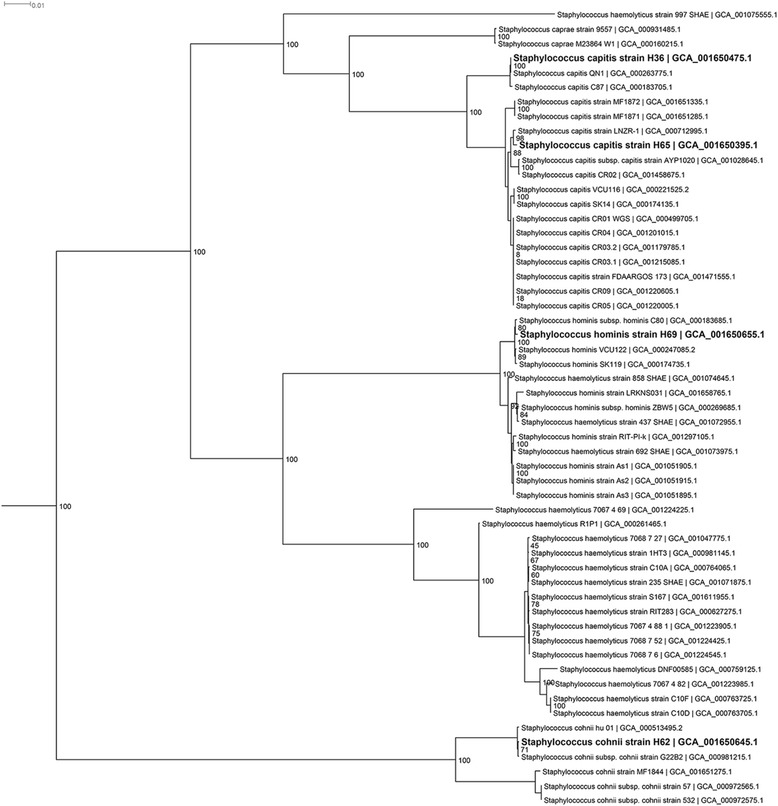
Maximum Likelihood tree based on concatenated markers from *Staphylococcus* spp. genomes. The tree was inferred using FastTree from an Hmmalign alignment in Phylosift of 37 highly conserved marker genes. Numbers at the nodes represent local support values. The tree was rooted to *Macrococcus caseolyticus* as an outgroup (not shown) since this species is a close relative to *Staphylococcus*

The concatenated marker genes for one isolate placed it in a well-supported clade of *P. oryzihabitans* isolates (Additional file 5) and thus we have named this *P. oryzihabitans* H72. Based on this tree, we believe that one of the (unpublished) strains of *P. psychrotolerans* has been misclassified and should also be considered *P. oryzihabitans*. Four of the isolates were *Staphylococcus* species, for which we created a single concatenated marker tree containing the relevant close relatives of the isolates (Fig. 1). Two of our *Staphylococcus* isolates placed within a well-supported (i.e., high bootstrap support) monophyletic clade of *S. capitis* strains and thus we have named these *S. capitis* H36 and *S. capitis* H65. One *Staphylococcus* isolate placed within a well-supported clade of *S. cohnii* strains and thus we have named it *S. cohnii* H62. Our fourth *Staphylococcus* isolate was placed within a well-supported clade containing mostly *S. hominis* isolates but which also contains a few *S. haemolyticus* isolates. Because this tree shows a distinct clade containing many *S. haemolyticus* isolates, we have named this isolate *S. hominis* H69. It is unclear from this tree alone whether these few *S. haemolyticus* isolates are misnamed or whether further taxonomic revision of this group is needed.

**Fig. 2:**
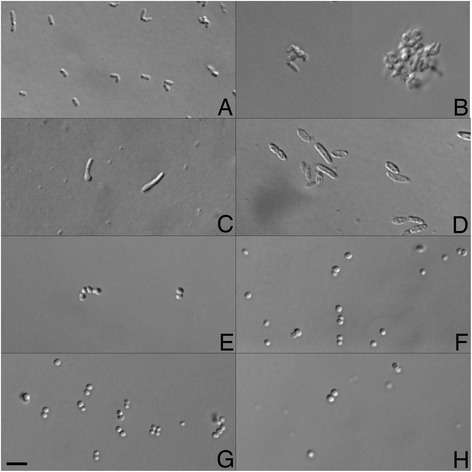
Transmitted light microscope images of the eight isolates. Bar is 5 μm. **a** Rod-shaped cells of *Microbacterium* sp. H83 **b** *Mycobacterium iranicum* H39; note, this organism was sparse in the images and tended to be highly clumped, so two snapshots were used for the sake of visualization **c** pleomorphic, rod-shaped cells of *Plantibacter* sp. H53 **d** *Pseudomonas oryzihabitans* H72, rods with rounded ends typically occurring as solitary cells but rarely also in pairs, **e** *Staphylococcus capitis* H36, occurring in pairs or strings of cells **f** *Staphylococcus capitis* H65, as single cells and pairs **g** *Staphylococcus cohnii* H62, as single cells, pairs, and occasionally threes or tetrads, **h** *Staphylococcus hominis* H69, as single cells and pairs. Images were collected using a Zeiss M1 AxioImager equipped with DIC and a Hamamatsu Orca 03 camera run by BioVision’s iVision software. Images were cropped and organized into a plate using Adobe Photoshop CS6

General description of the isolates are summarized in Table 1, and micrographs appear in Fig. 2.

**Table 1:** Classification and general features of the eight isolates in accordance with the MIGS recommendations [60]

MIGS ID	Property	*Microbacterium* sp. H83	Evidence code^a^	*Mycobacterium iranicum* H39	Evidence code	*Plantibacter* sp. H53	Evidence code	*Pseudomonas oryzihabitans* H72	Evidence code	*Staphylococcus capitis* H36	Evidence code	*S. capitis* H65	Evidence code	*S.cohnii* H62	Evidence code	*S. hominis* H69	Evidence code
	Classification																
	Domain	Bacteria	TAS [61]	Bacteria	TAS [61]	Bacteria	TAS [61]	Bacteria	TAS [61]	Bacteria	TAS [61]	Bacteria	TAS [61]	Bacteria	TAS [61]	Bacteria	TAS [61]
	Phylum	*Actinobacteria*	TAS [62]	*Actinobacteria*	TAS [62]	*Actinobacteria*	TAS [62]	*Proteobacteria*	TAS [63]	*Firmicutes*	TAS [64]	*Firmicutes*	TAS [64]	*Firmicutes*	TAS [64]	*Firmicutes*	TAS [64]
	Class	*Actinobacteria*	TAS [65]	*Actinobacteria*	TAS [65]	*Actinobacteria*	TAS [65]	*Gammaproteobacteria*	TAS [66, 67]	*Bacilli*	TAS [68, 69]	*Bacilli*	TAS [68, 69]	*Bacilli*	TAS [68, 69]	*Bacilli*	TAS [68, 69]
	Order	*Micrococcales*	TAS [70, 71]	*Corynebacteriales*	TAS [72, 73]	*Micrococcales*	TAS [70, 71]	*Pseudomonadales*	TAS [71, 74]	*Bacillales*	TAS [70, 71]	*Bacillales*	TAS [70, 71]	*Bacillales*	TAS [70, 71]	*Bacillales*	TAS [70, 71]
	Family	*Microbacteriaceae*	TAS [75, 76]	*Mycobacteriaceae*	TAS [71, 77]	*Microbacteriaceae*	TAS [75, 76]	*Pseudomonadaceae*	TAS [71, 78]	Staphylococcaceae	TAS [79]	Staphylococcaceae	TAS [79]	Staphylococcaceae	TAS [79]	Staphylococcaceae	TAS [79]
	Genus	*Microbacterium*	TAS [71, 80]	*Mycobacterium*	TAS [71, 81]	*Plantibacter*	TAS [15]	*Pseudomonas*	TAS [71, 82]	*Staphylococcus*	TAS [71, 83]	*Staphylococcus*	TAS [71, 83]	*Staphylococcus*	TAS [71, 83]	*Staphylococcus*	TAS [71, 83]
	Species	*Microbacterium* sp.	NAS	*M. iranicum*	TAS [11]	*Plantibacter* sp.	NAS	*P. oryzihabitans*	TAS [84]	*S. capitis*	TAS [71, 85]	*S. capitis*	TAS [71, 85]	*S. cohnii*	TAS [71, 86]	*S. hominis*	TAS [71, 85]
	Strain	H83	IDA	H39	IDA	H53	IDA	H72	IDA	H36	IDA	H65	IDA	*H62*	IDA	H69	IDA
	Gram stain	Positive	TAS [23]	Positive	TAS [11]	Positive	TAS [87]	Negative	TAS [30]	Positive	TAS [88]	Positive	TAS [88]	Positive	TAS [88]	Positive	TAS [88]
	Cell shape	Rod	TAS [23]	Rod	TAS [11]	Rod	TAS [87]	Rod	TAS [30]	Coccus/grape-like clusters	TAS [88]	Coccus/grape-like clusters	TAS [88]	Coccus/grape-like clusters	TAS [88]]	Coccus/grape-like clusters	TAS [88]
	Motility	nd		Non-motile	TAS [11]	Non-motile	TAS [87]	Motile	TAS [30]	Non-motile	TAS [88]	Non-motile	TAS [88]	Non-motile	TAS [88]	Non-motile	TAS [88]
	Sporulation	Non-spore forming	TAS [23]	Non-spore forming	TAS [11]	Non-spore forming	TAS [87]	Non-spore forming	NAS	Non-spore forming	TAS [88]	Non-spore forming	TAS [88]	Non-spore forming	TAS [88]]	Non-spore forming	TAS [88]
	Temperature range	Mesophile	TAS [23]	25-40°	TAS [11]	Mesophile	IDA	Mesophile	TAS [30]	18-45 °C	TAS [88]	18-45 °C	TAS [88]	15-45 °C	TAS [88]	20-45 °C	TAS [88]
	Optimum temperature	nd		37°	TAS [11]	30 °C	TAS [87]	nd		nd	IDA	nd		nd		30-40 °C	TAS [88]
	pH range; Optimum	5-11; nd	TAS [23]	nd		nd		nd		nd		nd		nd		nd	
	Carbon source	Yeast extract, Peptone, Dextrose, Starch, Casamino acids	IDA	Yeast extract, Peptone, Dextrose, Starch, Casamino acids	IDA	Yeast extract, Peptone, Dextrose, Starch, Casamino acids	IDA	Yeast extract, Peptone, Dextrose, Starch, Casamino acids	IDA	Yeast extract, Peptone, Dextrose, Starch, Casamino acids	IDA	Yeast extract, Peptone, Dextrose, Starch, Casamino acids	IDA	Yeast extract, Peptone, Dextrose, Starch, Casamino acids	IDA	Yeast extract, Tryptone	IDA
GS-6	Habitat	Indoor air	NAS	Indoor air	NAS	Indoor air	NAS	Indoor air	NAS	Indoor air	NAS	Indoor air	NAS	Indoor air	NAS	Indoor air	NAS
6.3	Salinity	Normal	IDA	5% NaCl (w/v)	TAS [11]	Normal	IDA	6.5% NaCl (w/v)	TAS [84]	10% NaCl (w/v)	TAS [88]	10% NaCl (w/v)	TAS [88]	10% NaCl (w/v)	TAS [88]	10% NaCl (w/v)	TAS [88]
22	Oxygen requirement	Aerobic	TAS [23]	Aerobic	TAS [89]	Aerobic	TAS [87]	Aerobic	TAS [30]	Facultative anaerobes	TAS [88]	Facultative anaerobes	TAS [88]	Facultative anaerobes	TAS [88]	Facultative anaerobes	TAS [88]
15	Biotic relationship	Free living	NAS	Symbiont	TAS [11]	Free living	TAS [87]	Free living; symbiont	TAS [84]	Free living	NAS	Free living	NAS	Free living	NAS	Free living	NAS
14	Pathogenicity	nd		nd		nd		nd		nd		nd		nd		nd	
4	Geographic location	USA: California: Piedmont	NAS	USA: California: Oakland	NAS	USA: California: Walnut Creek	NAS	USA: California: Oakland	NAS	USA: California: Oakland	NAS	USA: California: Milpitas	NAS	USA: California: Milpitas	NAS	USA: California: Milpitas	NAS
5	Sample collection	2015-03-16	NAS	2015-03-18	NAS	2015-03-17	NAS	2015-03-18	NAS	2015-03-18	NAS	2015-03-31	NAS	2015-03-31	NAS	2015-03-31	NAS
4.1	Latitude	37°49'25.6"	NAS	122°16'21.9"	NAS	122°03'50.1"	NAS	122°16'21.9"	NAS	122°16'21.9"	NAS	121°53'59.0"	NAS	121°53'59.0"	NAS	121°53'59.0"	NAS
4.2	Longitude	122°13'53.9"	NAS	37°48'41.1"	NAS	37°54'49.4"	NAS	37°48'41.1"	NAS	37°48'41.1"	NAS	37°25'57.7"	NAS	37°25'57.7"	NAS	37°25'57.7"	NAS
4.4	Altitude	100 m	NAS	13 m	NAS	40 m	NAS	13 m	NAS	13 m	NAS	6 m	NAS	6 m	NAS	6 m	NAS

^a^Evidence codes – *IDA* inferred from direct assay, *TAS* traceable author statement, *NAS* non-traceable author statement, *nd* not determined. These evidence codes are from the Gene Ontology project [90]

*Staphylococcus* are non-spore-forming, non-motile round-shaped cells (Fig. 2 e-h). They demonstrate habitat preference in the human body with *S. capitis* mainly being found on the adult head and *S. cohnii* on the feet [10]. *S. hominis* is the main colonizer of head, axillae, arms, and legs, and is frequently encountered in nosocomial infections.

Organisms within the genus *Microbacterium* spp. are yellow-pigmented, aerobic, rod-shaped, Gram-positive bacteria [23] (Fig. 2a). They have been isolated from numerous and variable environments, including soil and water [24], the phyllosphere [25], human patients [26], and a residential toilet [27], and they have been associated with endophthalmitis [28] and catheter infections [29].

*Pseudomonas oryzihabitans* (Fig. 2d) is a Gram-negative, non-fermenting, yellow-pigmented bacterium [30]. Despite its environmental origin, *P. oryzihabitans* has been recognized as a potential pathogen in recent years, especially in immunocompromised hosts, both in nosocomial or community-level settings. It can form biofilms in aquifers in association with suspended particulate matter, which can be subsequently entrained into the drinking water distribution systems, posing a potential risk for human health given their resistance to chlorine compared to their planktonic counterparts [13]. This species has been associated with catheter [31] and bloodstream infections, endophthalmitis [32], necrotic enteritis [33], and peritonitis ([34] and references therein). There are two instances in which the source of human infection has been well documented, and the source has been found to be a synthetic sponge, one used by an immunocompromised individual [31] and another in the milk kitchen of a neonatal intensive care unit [33].

*Mycobacterium iranicum* (Fig. 2b) is a newly described, rapidly growing, orange-pigmented scotochromogenic, non-tuberculous mycobacterial species. Its clinical significance is still under study but it has been associated with patients with pulmonary infections, such as pneumonia, chronic obstructive airway disease, and bronchiectasis [11, 35].

Lastly, *Plantibacter* (Fig. 2c) are pleomorphic, rod-shaped, yellow-pigmented, aerobic, Gram-positive bacteria that belong to the class of *Actinobacteria*.

## Genome sequencing information

### Genome project history

These genomes were generated as part of a project to sequence reference genomes from the built Environment, funded by the Alfred P. Sloan Foundation through their “Microbiology of the Built Environment” Program. Sequencing and assembly of all isolates were performed at the University of California, Davis. The genome sequences were deposited in GenBank and given a Genome On-Line Database identifier [36]. Project information and association with MIGS version 2.0 are presented in Table 2.

**Table 2:** Project information

MIGS ID	Property	*Microbacterium* sp. H83	*Mycobacterium iranicum* H39	*Plantibacter* sp. H53	*Pseudomonas oryzihabitans* H72	*Staphylococcus capitis* H36	*S. capitis* H65	*S. cohnii* H62	*S. hominis* H69
MIGS 31	Finishing quality	Permanent Draft	Permanent Draft	Permanent Draft	Permanent Draft	Permanent Draft	Permanent Draft	Permanent Draft	Permanent Draft
MIGS-28	Libraries used	Illumina PE library (300 bp insert size)	Illumina PE library (300 bp insert size)	Illumina PE library (300 bp insert size)	Illumina PE library (300 bp insert size)	Illumina PE library (300 bp insert size)	Illumina PE library (300 bp insert size)	Illumina PE library (300 bp insert size)	Illumina PE library (300 bp insert size)
MIGS 29	Sequencing platforms	Illumina MiSeq	Illumina MiSeq	Illumina MiSeq	Illumina MiSeq	Illumina MiSeq	Illumina MiSeq	Illumina MiSeq	Illumina MiSeq
MIGS 31.2	Fold coverage	239x	95x	115x	112x	258x	170x	157x	64x
MIGS 30	Assemblers	A5-miseq	A5-miseq	A5-miseq	A5-miseq	A5-miseq	A5-miseq	A5-miseq	A5-miseq
MIGS 32	Gene calling method	IMG	IMG	IMG	IMG	IMG	IMG	IMG	IMG
	Locus Tag	A4X16	A4X20	A4X17	A4X15	A4X14	A4X13	A4A82	A3836
	Genbank ID	LWCU01000000.1	LWCS01000000.1	LWCT01000000.1	LWCR01000000.1	LWCQ01000000.1	LWCP01000000.1	LWAC01000000.1	LVVO01000000.1
	Genbank Date of Release	May 24, 2016	May 24, 2016	May 24, 2016	May 24, 2016	May 24, 2016	May 24, 2016	May 24, 2016	May 24, 2016
	GOLD ID	Gp0147178	Gp0147183	Gp0147185	Gp0147186	Gp0147187	Gp0147188	Gp0147192	Gp0147190
	BIOPROJECT	PRJNA317658	PRJNA317657	PRJNA317656	PRJNA317602	PRJNA317600	PRJNA317599	PRJNA316869	PRJNA316465
MIGS 13	Source Material Identifier	DSM-103506	DSM-103542	DSM-103507	DSM-103505	DSM-103511	DSM-103512	DSM-103510	DSM-103553
	Project relevance	Built Environment Reference Genomes							

### Growth conditions and genomic DNA preparation

Strains were initially collected through environmental sampling (see Classification and features section) and were subsequently deposited into the DMSZ. Glycerol stocks of all isolates were initially grown at 28 °C on LB plates. A single colony was then inoculated in LB and incubated at 28 °C for 18 h (except for *M. iranicum* strain H39, grown at 37 °C for 5 days). DNA was subsequently extracted from the cultures using the DNeasy Blood and Tissue kit (Qiagen), and the quality was assessed using a NanoDropTM spectrophotometer.

### Genome sequencing and assembly

Barcoded Illumina paired-end libraries were generated from all samples using the Nextera XT kit (Illumina). After pooling, the libraries were size-selected for a range of 600–900 bp on a Pippin Prep (Sage Science) and then sequenced on an Illumina MiSeq (Paired End 300 bp). After demultiplexing with a custom script, the reads from each sample were assembled using the A5-miseq pipeline, which automates the process of adapter removal, quality trimming, error-correction, and contig generation [37, 38]. The completeness and contamination of the assemblies was estimated using PhyloSift [22] and CheckM [39]. Across all strains, genome completeness was determined to be a minimum of 98.9%, and the maximum contamination was 0.99% (Additional file 1).

### Genome annotation

Isolates were predominantly annotated using the IMG system [40] with no additional manual curation. Table 3 summarizes genome statistics and Table 4 the COG functional categories for the eight isolates according to IMG. Additional annotations were performed with PGAP [41] and RAST [42]. The full-length 16S rRNA gene sequences for each isolate, used for tree building (see above), were extracted from RAST.

**Table 3:** Genome statistics

	*Microbacterium* sp. H83		*Mycobacterium iranicum* H39		*Plantibacter* sp. H53		*Pseudomonas oryzihabitans* H72		*Staphylococcus capitis* H36		*S. capitis* H65		*S. cohnii* H62		*S. hominis* H69	
Attribute	Value	%^a^	Value	%	Value	%	Value	%	Value	%	Value	%	Value	%	Value	%
Genome size (bp)	3,531,197	100	6,470,840	100	4,012,045	100	5,316,471	100	2,412,840	100	2,482,551	100	2,656,939	100	2,335,200	100
DNA coding (bp)	3,256,592	92	5,997,369	93	3,678,027	92	4,723,080	89	2,064,637	86	2,146,765	86	2,213,412	83	2,024,342	87
DNA G + C (bp)	2,459,099	70	4,277,463	66	2,783,137	69	3,459,720	65	789,696	33	812,206	33	859,087	32	733,146	31
DNA scaffolds/contigs	52	100	91	100	50	100	78	100	24	100	31	100	262	100	143	100
Total genes	3522	100	6227	100	3826	100	5005	100	2454	100	2476	100	2761	100	2450	100
Protein coding genes	3462	98	6162	99	3763	98	4897	98	2355	96	2378	96	2666	97	2350	96
RNA genes	60	2	65	1	63	2	108	2	99	4	98	4	95	3	100	4
Pseudo genes	0	0	0	0	0	0	0	0	0	0	0	0	0	0	0	0
Genes in internal clusters	874	25	2189	35	1075	28	1487	30	451	18	456	18	593	21	464	19
Genes with function prediction	2637	75	4691	75	2943	77	3969	79	1970	80	1970	80	2151	78	1907	78
Genes assigned to COGs	2271	64	3929	63	2567	67	3605	72	1759	72	1802	73	1877	68	1708	70
Genes with Pfam domains	2783	79	4989	80	3093	81	4209	84	2044	83	2057	83	2231	81	1979	81
Genes with signal peptides	148	4	366	6	164	4	496	10	67	3	70	3	55	2	95	3
Genes with transmembrane helices	953	27	1365	22	1117	29	1138	23	637	26	632	26	671	24	575	23
CRISPR repeats	0	0	0	0	0	0	0	0	0	0	0	0	0	0	0	0

^a^The percentage of total is based on either the size of the genome in base pairs or the total number of genes in the annotated genome

**Table 4:** Numbers of genes associated with general COG functional categories

		*Microbacterium* sp. H83		*Mycobacterium iranicum* H39		*Plantibacter* sp. H53		*Pseudomonas oryzihabitans* H72		*Staphylococcus capitis* H36		*S. capitis* H65		*S. cohnii* H62		*S. hominis* H69	
Code	Description	Value	%^a^	Value	%	Value	%	Value	%	Value	%	Value	%	Value	%	Value	%
J	Translation, ribosomal structure and biogenesis	167	4.8	191	3.1	172	4.6	242	4.9	188	8.0	191	8.0	192	7.2	187	8.0
A	RNA processing and modification	1	0.0	1	0.0	1	0.0	1	0.0	0	0.0	0	0.0	0	0.0	0	0.0
K	Transcription	265	7.7	403	6.5	306	8.1	328	6.7	125	5.3	132	5.6	141	5.3	128	5.4
L	Replication, recombination and repair	109	3.1	120	1.9	105	2.8	134	2.7	86	3.7	95	4.0	89	3.3	101	4.3
B	Chromatin structure and dynamics	0	0.0	0	0.0	0	0.0	2	0.0	1	0.0	1	0.0	1	0.0	0	0.0
D	Cell cycle control, Cell division, chromosome partitioning	24	0.7	33	0.5	24	0.6	38	0.8	24	1.0	26	1.1	25	0.9	27	1.1
V	Defense mechanisms	50	1.4	118	1.9	71	1.9	80	1.6	50	2.1	41	1.7	49	1.8	38	1.6
T	Signal transduction mechanisms	87	2.5	189	3.1	121	3.2	286	5.8	67	2.8	68	2.9	65	2.4	66	2.8
M	Cell wall/membrane biogenesis	115	3.3	222	3.6	140	3.7	245	5.0	96	4.1	101	4.2	104	3.9	108	4.6
N	Cell motility	31	0.9	11	0.2	9	0.2	153	3.1	6	0.3	8	0.3	5	0.2	4	0.2
U	Intracellular trafficking and secretion	28	0.8	22	0.4	17	0.5	77	1.6	19	0.8	20	0.8	14	0.5	18	0.8
O	Posttranslational modification, protein turnover, chaperones	97	2.8	138	2.2	92	2.4	161	3.3	75	3.2	78	3.3	78	2.9	74	3.1
C	Energy production and conversion	141	4.1	331	5.4	128	3.4	243	5.0	111	4.7	111	4.7	109	4.1	99	4.2
G	Carbohydrate transport and metabolism	270	7.8	244	4.0	392	10.4	275	5.6	131	5.6	133	5.6	150	5.6	118	5.0
E	Amino acid transport and metabolism	274	7.9	337	5.5	304	8.1	403	8.2	174	7.4	188	7.9	202	7.6	170	7.2
F	Nucleotide transport and metabolism	77	2.2	98	1.6	82	2.2	91	1.9	79	3.4	78	3.3	82	3.1	80	3.4
H	Coenzyme transport and metabolism	145	4.2	295	4.8	156	4.1	207	4.2	136	5.8	136	5.7	125	4.7	126	5.4
I	Lipid transport and metabolism	113	3.3	478	7.8	134	3.6	168	3.4	91	3.9	88	3.7	93	3.5	78	3.3
P	Inorganic ion transport and metabolism	166	4.8	244	4.0	171	4.5	261	5.3	136	5.8	143	6.0	141	5.3	136	5.8
Q	Secondary metabolites biosynthesis, transport and catabolism	58	1.7	322	5.2	65	1.7	94	1.9	40	1.7	43	1.8	46	1.7	36	1.5
R	General function prediction only	233	6.7	571	9.3	285	7.6	348	7.1	183	7.8	184	7.7	197	7.4	164	7.0
S	Function unknown	107	3.1	224	3.6	138	3.7	217	4.4	139	5.9	143	6.0	156	5.9	128	5.4
-	Not in COGs	1251	36.1	2298	37.3	1259	33.5	1400	28.6	695	29.5	674	28.3	884	33.2	742	31.6

^a^ Percent of annotated genes. The total is based on the total number of protein coding genes in the genome

## Genome properties

Genome sizes were smallest for the *Staphylococcus* isolates at approximately 2.5 Mbps and largest for *M. iranicum* H39 at nearly 6.5 Mbps (Table 3). Similarly, the DNA G + C content was lowest for the *Staphylococcus* isolates (approximately 31%) and much higher for the other four isolates (at least 65% content). Predicted coding regions accounted for 83–93% of the genomes for all eight isolates, and the total number of predicted genes ranged from 2450 in *S. hominis* H69 to 6227 in *M. iranicum* H39. The percentage of genes with a functional prediction was fairly consistent across the genomes, ranging from 75 to 80%. The percentage of RNA genes for the *Staphylococcus* isolates ranged from 3 to 4% and were higher than the others isolates (1–2%). Conversely, the percentage of genes in internal clusters (an indicator of non-redundant sequences) ranged from 18 to 21% in the *Staphylococcus* isolates but ranged from 25 to 35% in the other isolates. The genome of *P. oryzihabitans* H72 encoded a much higher percentage of signal peptides than the other genomes (Tables 3 and 4). Neither pseudogenes nor CRISPR repeats were identified in any of the genomes.

For all strains, 27–37% of the proteins were not predicted to be part of a COG category (Table 4). *P. oryzihabitans* was the only recognized motile organism (Table 1), and *P. oryzihabitans* H72 showed a much greater percentage of genes related to motility (Table 4). *M. iranicum* H39 harbored a much higher percentage of genes for the COG categories of lipid transport/metabolism and secondary metabolites biosynthesis/transport/catabolism than the other isolates. There was no observed relationship between genome coverage (Table 2) and the percentage of unassigned proteins (Table 4).

## Insights from the genome sequences

### Phylogenetic comparisons

The genomes of the sequenced isolates were compared to publicly available closely related genomes to determine the ANI values [43]. For those six isolates in which a species epithet was given based on gene trees, ANI values were greater than 90% (Additional file 6), and were greater than 96% for the *Staphylococcus* isolates. The genomes of those isolates that were assigned to genera based on gene trees were compared to closely related publicly available genomes. For *Microbacterium* sp. H83, the ANI value with *M. hydrocarbonoxydans* was 84.1% and for *Plantibacter* sp. H53 was 87.8% with another *Plantibacter* sp. (Additional file 6).

### Virulence and biofilm production

CoNS are opportunistic pathogens and they do not encode for virulence factors (e.g., exotoxins) commonly found in pathogenic species such as *S. aureus*. However, they do encode genes related to biofilm formation, persistence and immune invasion [44]. The attachment to a surface is the first step to successful colonization and a precursor for the establishment of infection. In the IMG annotation, we found genes with predicted functions to be associated with cell wall-associated FBP, such as fbe, and several other surface-associated proteins such as a bifunctional autolysin and putative adhesins. However, the gene fbe was not found in *S. capitis* H36, and another gene known to be important for surface adhesion in *Staphylococcus*, ebh [44], was not observed in any isolate. Both Ebh and FBP act as adhesins but FBP also acts as an invasin, facilitating binding and internalization in host cells [45]. Additionally, we found genes with predicted functions to be associated with Microbial Surface Components Recognizing Adhesive Matrix Molecules, such as the sdrG gene. Further biofilm accumulation is mediated by exopolysaccharides such as PNAG and PGA. Genes related only to PGA (*cap* operon), which have been shown to provide resistance to phagocytosis and to a host’s antimicrobial peptides in *S. epidermidis* [46], were identified. Genes encoding predicted pro-inflammatory molecules with cytolytic and antimicrobial properties such as β-type phenol soluble modulins (PSM) [44] were found in all four staphylococci strains, along with genes encoding their accessory regulator B (Agr) [47]. Other systems important for the regulation of virulence in staphylococci that were found in our strains included the staphylococcal accessory regulator Sar, one of the two components of each of the regulatory systems, SaeRS and ArlRS, and an infection-related protease, ClpC [44].

### Antibiotic resistance

We used the Resistance Gene Identifier of CARD [48] to explore possible genes related to antimicrobial resistance. Microbial genome sequencing has the potential to be used as a prediction tool of antibiotic resistance in clinical settings [49, 50], and in fact has been shown to be a promising approach in *S. aureus* [51, 52] as well as other bacteria [53]. However, at the moment, clinical testing of antibiotic resistance is restricted to PCR-based targeting of specific genes [54, 55], and many of the genes in antibiotic databases have not been verified in clinical settings and are subject to errors in annotation (e.g., [56]). Nevertheless, we surveyed genes predicted to confer antibiotic resistance in order to explore commonalities across the different isolates. Additional file 7 details the Gene ID and other information stemming from the IMG annotation of putative antibiotic resistance genes identified in CARD. Limiting the results to “perfect” and “strict” hits, many of these genes included efflux pumps predicted to confer resistance to more than one class of antimicrobials (e.g., fluoroquinolones, tetracyclines, polymyxins) as well as genes predicted to be associated with resistance to specific antimicrobials (e.g., beta-lactams, aminocoumarins, chloramphenicol, aminoglycosides, and fosfomycin). Some antimicrobial genes were common to many strains; others were limited to specific taxonomic groups. For example, all eight strains were found to contain genes predicted to confer resistance to mupirocin and fosfomycin, while genes for fusidic acid resistance were only observed in *S. capitis* H65 (Additional file 7).

In addition to general targeting of antibiotic resistance genes, we also looked specifically for genes related to triclosan resistance. TCS is a synthetic antimicrobial agent that is commonly used in home and personal care products such as hand soaps, toothpastes, deodorants, body washes, hand creams, body lotions, and cosmetics. It has been directly associated with the development of multidrug antibiotic resistance in a variety of primarily pathogenic bacteria via in vitro assays [57]. TCS induces resistance through mutations in the gene (*fab*I) that encodes TCS’s target enzyme (enoyl-acyl carrier protein reductase FabI) through overexpression, or through efflux pumps, with the latter only to be associated with multi-antibiotic resistance [57]. The *fab*I gene was identified only in one out of four staphylococci isolates, *S. capitis* H65, as well as in the *M. iranicum* H39 and *P. oryzihabitans* H72 genomes. We found several genes related to non-specific multidrug efflux pumps, such as *mex* genes (*mex*JKL) in their genomes. The MexJK efflux pump can efflux triclosan, but also requires the outer membrane protein channel composed of the OprM in order to efflux other antibiotics in *Pseudomonas aeruginosa* [58]*.* MexJK-OprM was found through CARD in all our genomes, except for *Plantibacter* sp. H53 that did not carry OprM. The triclosan efflux transporter TriABC–OpmH [59] was only partially present in *P. oryzihabitans* H72 (TriB was absent). Additionally, *P. oryzihabitans* H72 was the only isolate to contain an efflux pump predicted to offer triclosan resistance (Additional file 7). Susceptibility to TCS or other antibiotics has not been experimentally tested for the strains described here.

## Conclusions

The genomes of these eight isolates of bacteria collected from a residential environment will be valuable tools for exploring the basic microbiology of indoor microbes (e.g., overexpression of genes targeted by drugs/antimicrobial agents, such as triclosan, can provide insight into the mode of action of antibiotics and the associated development of resistance) as well as interpreting future metagenomic and transcriptomic datasets. These isolates represent seven species across five genera and likely originate from the dominant sources of indoor bacteria: the outdoor environment, human commensals, and premise plumbing.

## Additional files

Additional file 1: Table S1. Additional strain information, including growth conditions and genome completeness/contamination. (XLSX 36 kb)
Additional file 2: Figure S1. Phylogenetic tree of *Microbacterium* sp. H83. (PDF 153 kb)
Additional file 3: Figure S2. Phylogenetic tree of *Plantibacter* sp. H53. (PDF 153 kb)
Additional file 4: Figure S3. Phylogenetic tree of *Mycobacterium iranicum* H39. (PDF 146 kb)
Additional file 5: Figure S4. Phylogenetic tree of *Pseudomonas oryzihabitans* H72. (PDF 111 kb)
Additional file 6: Table S2. ANI values between these eight strains and selected genomes in the IMG database. (XLSX 8 kb)
Additional file 7: Table S3. Antibiotic genes detected in the eight genomes using CARD. (XLSX 47 kb)

## Abbreviations

ANI: Average nucleotide identity
BLAST: Basic local alignment search tool
CARD: Comprehensive antibiotic resistance database
COG: Clusters of orthologous groups
CoNS: Coagulase-negative staphylococci
FBP: Fibronectin/fibrinogen binding protein
IMG: Integrated microbial genomes
Pfam: Protein families
PGA: Poly-γ-glutamate
PNAG: Poly-N-acetylglucosamine
RAST: Rapid annotation using subsystem technology
TCS: Triclosan
