# Supplementary figures and images for: Draft genome sequences of eight bacteria isolated from the indoor environment: Staphylococcus capitis strain H36, S. capitis strain H65, S. cohnii strain H62, S. hominis strain H69, Microbacterium sp. strain H83, Mycobacterium iranicum strain H39, Plantibacter sp. strain H53, and Pseudomonas oryzihabitans strain H72
Source: Stand Genomic Sci. 2017 Jan 31;12:17. doi: 10.1186/s40793-017-0223-9 (PMC5282799; doi:10.1186/s40793-017-0223-9)

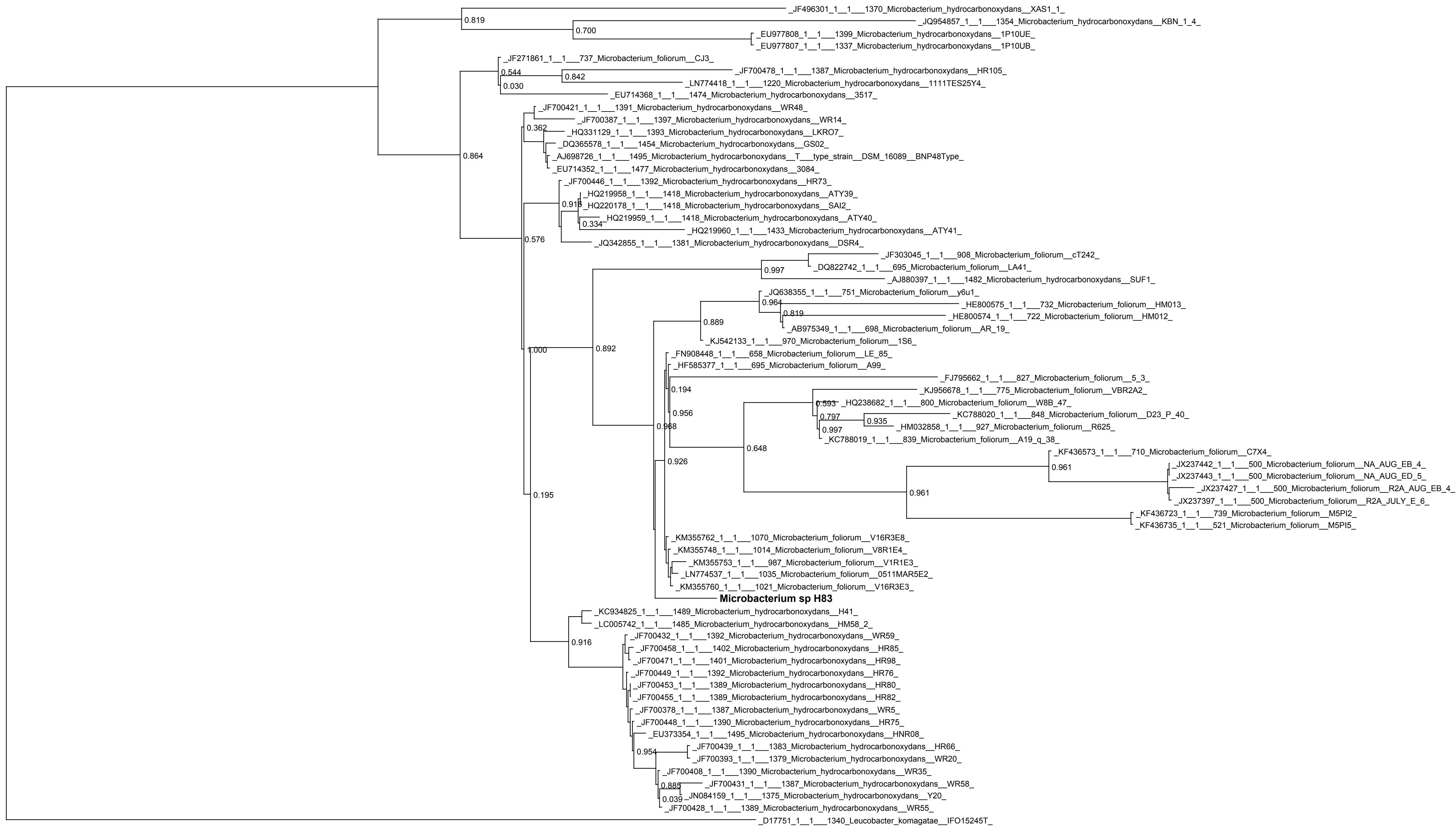

Supplement: Additional file 2: Figure S1. — Phylogenetic tree of Microbacterium sp. H83. (PDF 153 kb) [file 40793_2017_223_MOESM2_ESM.pdf]

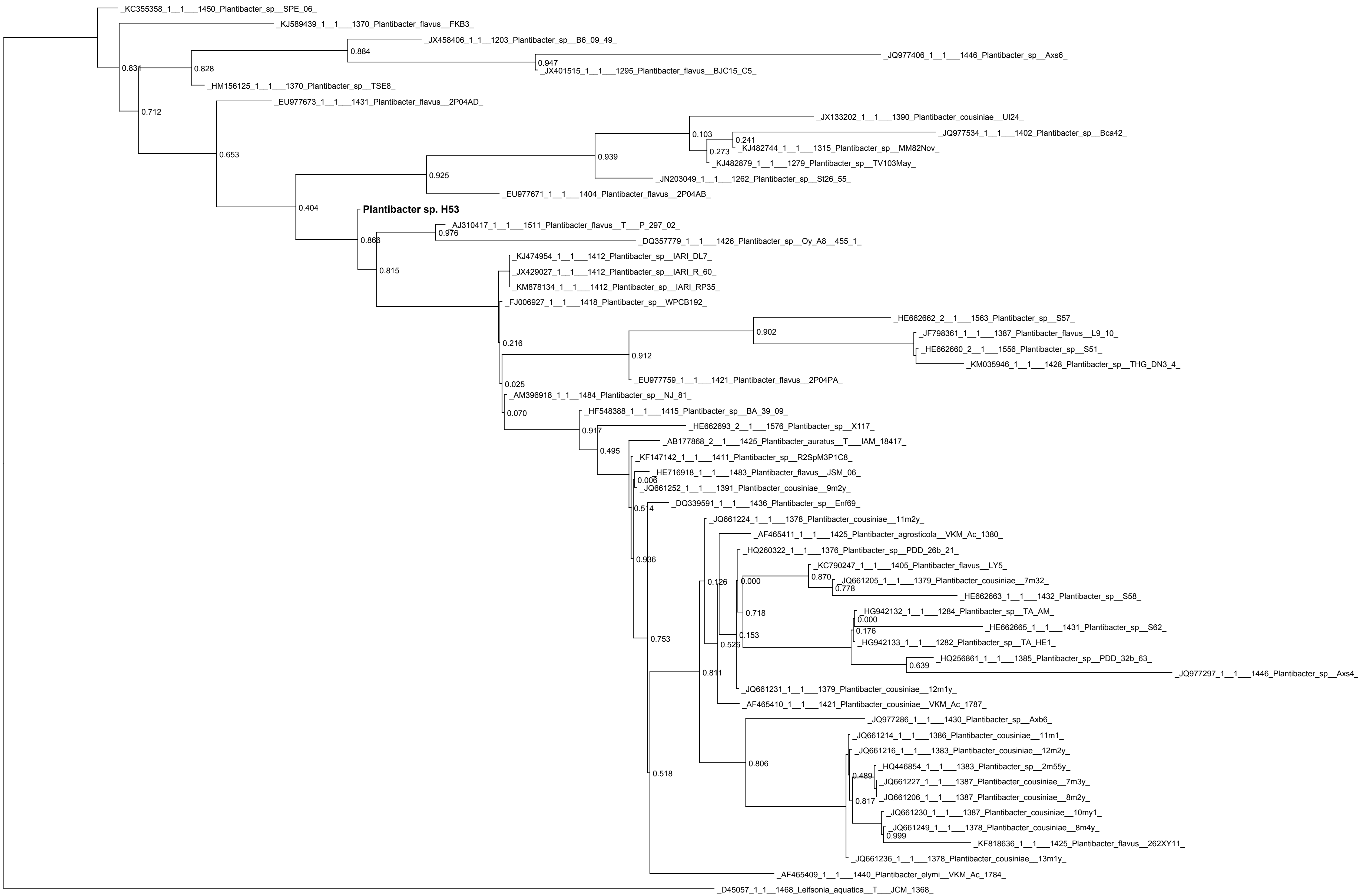

Supplement: Additional file 3: Figure S2. — Phylogenetic tree of Plantibacter sp. H53. (PDF 153 kb) [file 40793_2017_223_MOESM3_ESM.pdf]

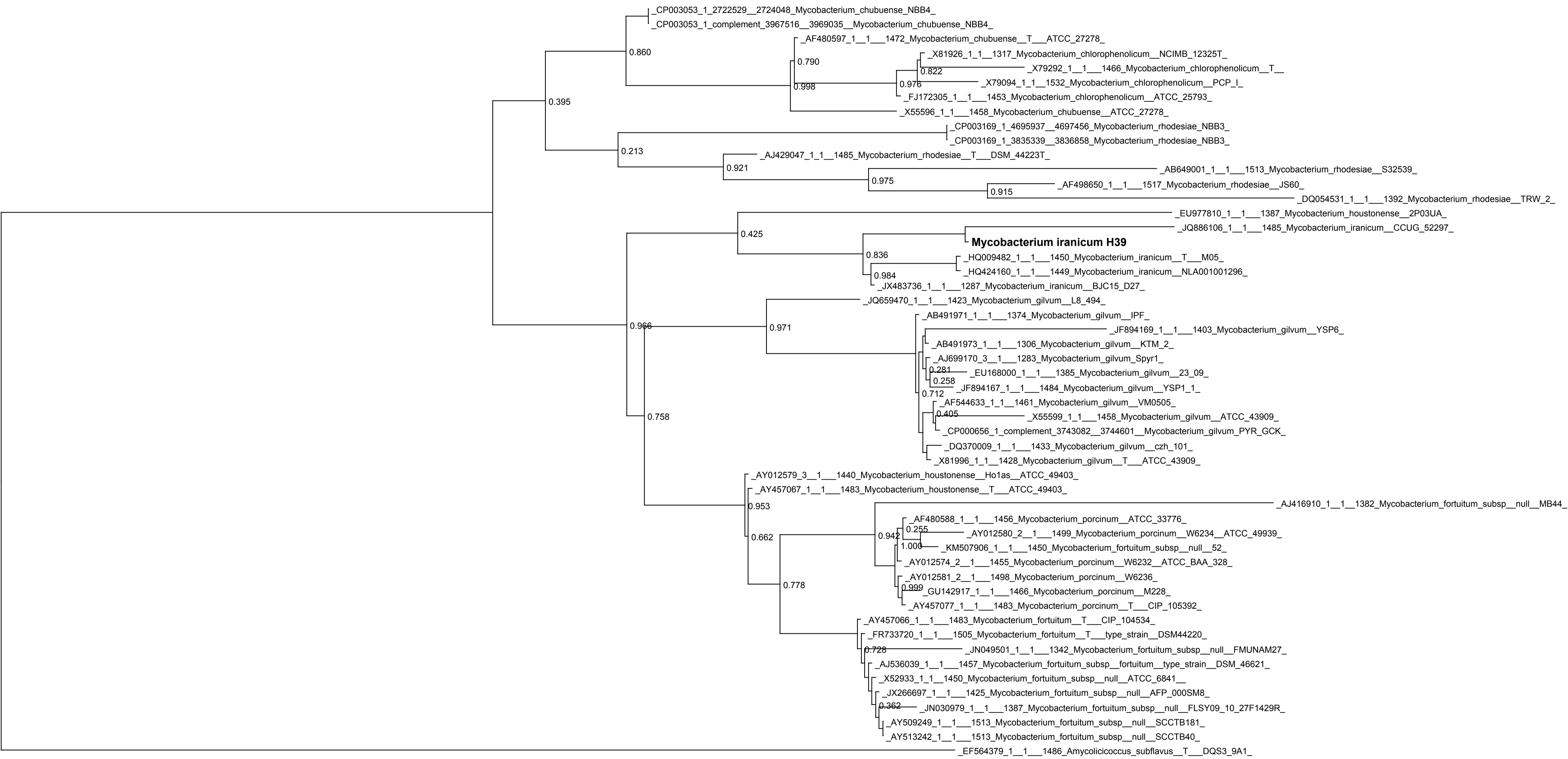

Supplement: Additional file 4: Figure S3. — Phylogenetic tree of Mycobacterium iranicum H39. (PDF 146 kb) [file 40793_2017_223_MOESM4_ESM.pdf]

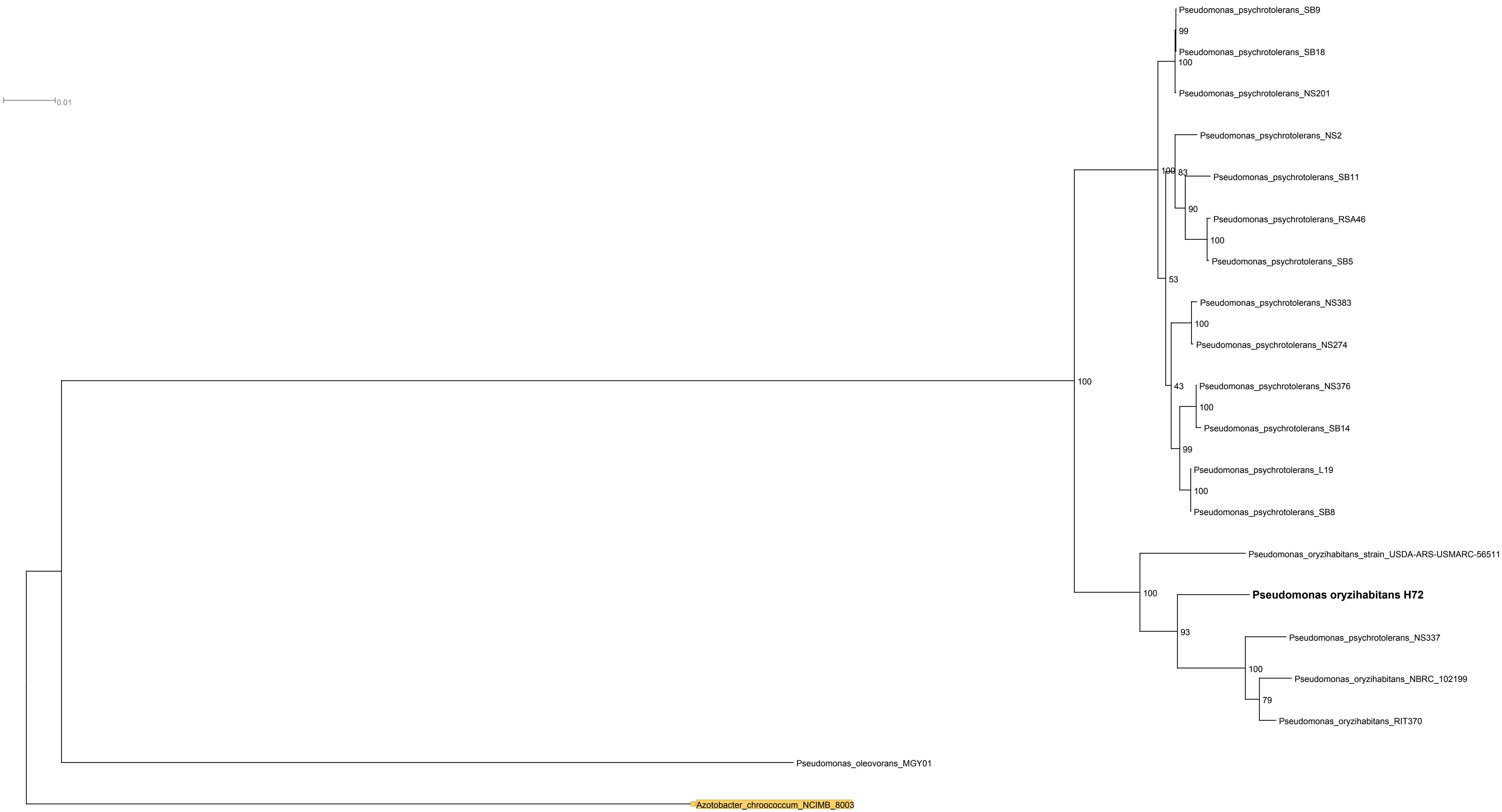

Supplement: Additional file 5: Figure S4. — Phylogenetic tree of Pseudomonas oryzihabitans H72. (PDF 111 kb) [file 40793_2017_223_MOESM5_ESM.pdf]
